# Cysteine as a Carbon Source, a Hot Spot in Cancer Cells Survival

**DOI:** 10.3389/fonc.2020.00947

**Published:** 2020-06-23

**Authors:** Jacinta Serpa

**Affiliations:** ^1^CEDOC, Chronic Diseases Research Centre, NOVA Medical School - Faculdade de Ciências Médicas, Universidade NOVA de Lisboa, Lisbon, Portugal; ^2^Instituto Português de Oncologia de Lisboa Francisco Gentil (IPOLFG), Lisbon, Portugal

**Keywords:** cysteine, cysteine metabolism, cysteine transport, cancer metabolic remodeling, targeting cysteine route

## Abstract

Cancer cells undergo a metabolic rewiring in order to fulfill the energy and biomass requirements. Cysteine is a pivotal organic compound that contributes for cancer metabolic remodeling at three different levels: (1) in redox control, free or as a component of glutathione; (2) in ATP production, via hydrogen sulfide (H_2_S) production, serving as a donor to electron transport chain (ETC), and (3) as a carbon source for biomass and energy production. In the present review, emphasis will be given to the role of cysteine as a carbon source, focusing on the metabolic reliance on cysteine, benefiting the metabolic fitness and survival of cancer cells. Therefore, the interplay between cysteine metabolism and other metabolic pathways, as well as the regulation of cysteine metabolism related enzymes and transporters, will be also addressed. Finally, the usefulness of cysteine metabolic route as a target in cancer treatment will be highlighted.

## Introduction

Posited as a glutathione precursor or as a source of sulfur and carbon, cysteine contributes for cancer cell strongness and prosperity, allowing their survival upon stressful microenvironmental conditions and upon drugs exposure (1, 2).

In the recent years, the role of cysteine and glutathione in the scavenging of reactive oxygen species (ROS), contributing for chemoresistance (3–9) have been under scrutiny. Cysteine and glutathione are crucial in the maintenance of the metabolic course (10–13), since the cancer metabolic rewiring implies the generation of oxidative stress (14–16). Nevertheless, cysteine has been underestimated as a carbon source, due to the core position of glycolysis in the cellular biosynthesis and bioenergetics, being major emphasis given to glucose as a preferential fuel and to glutamine as its main substitute [as reviewed in (17, 18)].

Despite few recent studies addressing cysteine as a key organic compound in cancer, the actual meaning of cancer cells' cysteine dependency is far from being completely known. Therefore, in the next sections, the metabolic dynamics of cysteine in cancer and the interconnections between cysteine metabolism and other metabolic pathways will be addressed.

## Cysteine as a Carbon Source in Cancer

The usefulness of cysteine as a carbon source is visible along the cysteine catabolic pathway, since cysteine catabolism originates organic compounds used in carbon and energy metabolism (19–23).

### Cysteine Metabolism and Other Metabolic Pathways Intercrosses

The metabolic reliance of cancer cells on cysteine promotes a better adaptation to metabolically damaging conditions and the development of chemoresistance (1, 2), accounting for cancer success.

**Figure 1:**
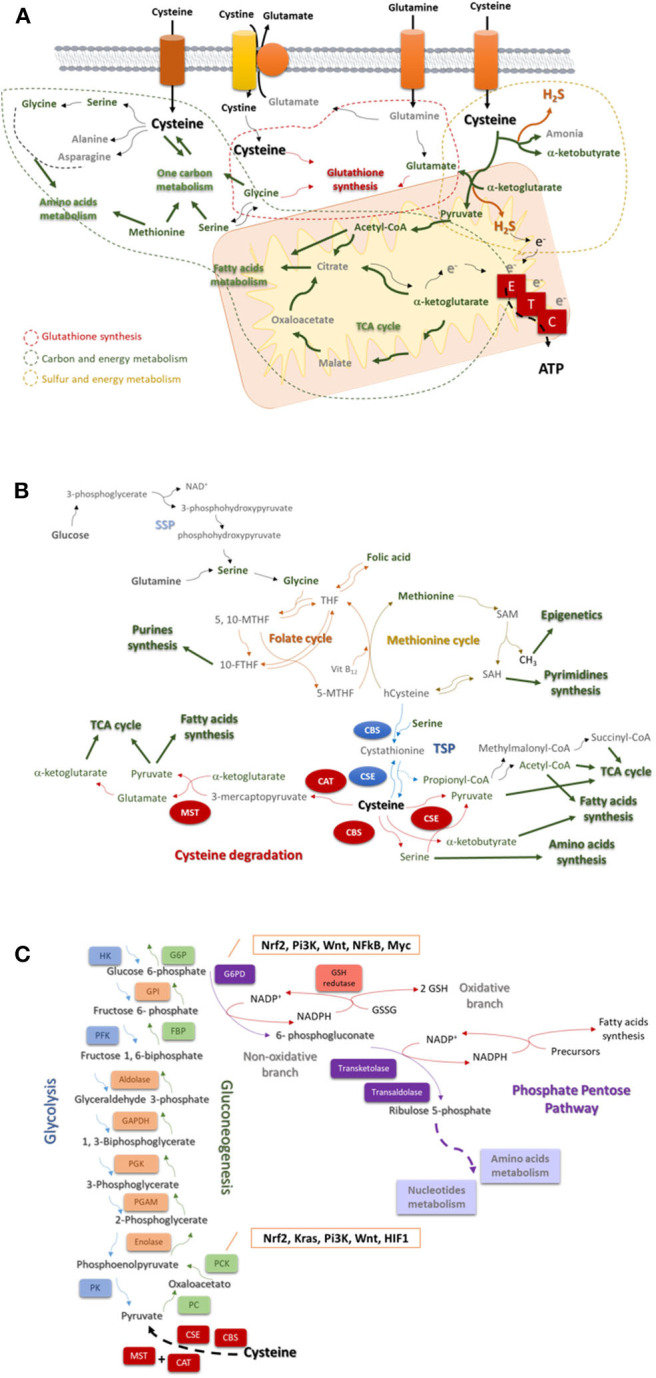
Cysteine is a core player in the cellular metabolism. **(A)** Cysteine is imported as cystine or as cysteine. Cysteine plays a pivotal role in cancer: it is incorporated in glutathione, a reactive oxygen species (ROS) scavenger; upon degradation in cytosol or in mitochondria, it supplies carbon and energy metabolism through FA and AA syntheses, tricarboxylic acid (TCA) cycle, one carbon metabolism and the production of ATP through the ETC, and it contributes for sulfur and energy production as a generator of hydrogen sulfide (H_2_S), a donor of electrons (e^−^) to ETC. **(B)** The one carbon metabolism is composed by the folate cycle and the methionine cycle. Serine, needed to start the folate cycle, can be glucose (serine synthesis pathway—SSP) or glutamine-originated. Serine originates glycine, which reacts with folic acid-derived tetrahydrofolate (THF), originating 5, 10-methylenetetrahydrofolate (5, 10-MTHF), which is converted into 5-methyltetrahydrofolate (5-MTHF) or 10-methyltetrahydrofolate (10-MTHF). 5-FTHF reacts with vitamin B_12_ (Vit B_12_) and homocysteine (hCysteine), forming THF and methionine. 10-FTHF is incorporated in the synthesis of purines, essential for nucleotides synthesis. In the methionine cycle, methionine is converted sequentially into S-adenosylmethionine (SAM), and to S-adenosylhomocysteine (SAH). The consequent release of a methyl group (CH_3_) will supply the methylation of DNA, DNA and histones. SAH is converted into hCysteine keeping on the methionine cycle, or it is deviated to the pyrimidines synthesis and consequently to nucleotides synthesis. Cysteine is *de novo* synthesized in the transsulfuration pathway (TSP), linking cysteine to the methionine cycle. The hCysteine, is converted into cystathionine through the condensation with serine. Cystathionine is hydrolyzed to cysteine and other organic compounds (e.g., α-ketoglutarate or propionate). Cysteine can be degraded and originate (directly or not) pyruvate, α-glutarate, α-ketobutyrate, serine, propionyl-CoA, succinate, and acetyl-CoA to supply the tricarboxylic acid (TCA) cycle, amino acids synthesis or the fatty acids synthesis. **(C)** Glycolysis is the degradation of a glucose molecule into 2 pyruvate molecules, through a sequence of reactions, having three irreversible steps catalyzed by hexokinase (HK), phosphofructokinase (PFK), and pyruvate kinase (PK). Gluconeogenesis is almost a reversion of glycolysis and cysteine-derived pyruvate is converted in glucose. The reversible steps are common to glycolysis and gluconeogenesis and are catalyzed by enolase, phosphoglycerate mutase (PGAM), phosphoglycerate kinase (PGK), Glyceraldehyde 3-phosphate dehydrogenase (GAPDH), aldolase, and Glucose-6-phosphate isomerase (GPI). The three irreversible steps of glycolysis impose gluconeogenesis to use four other enzymes: PC, pyruvate carboxylase; PKC, phosphoenolpyruvate carboxykinase; FBP, fructose 1,6-bisphosphatase; and G6PC, glucose 6-phosphatase. Gluconeogenesis is regulated by Nrf2, Kras, Pi3K, Wnt, and HIF1. Besides being an intermediate of glycolysis and gluconeogenesis glucose 6-phosphate is the substrate of phosphate pentose pathway (PPP), which has two biochemical branches (an oxidative and a non-oxidative branch) of reversible reactions. The non-oxidative branch of PPP uses glucose-6-phosphate to generate ribulose5-phosphate for AA and nucleotides synthesis. While the oxidative branch of PPP generates NADPH, involving the action of glutathione (GSH) reductase and the interplay with reductive biosynthesis, namely FA synthesis. PPP is regulated by Nrf2, Pi3K, Wnt, NFkB, and Myc.

Cysteine catabolism occurs upon the action of four enzymes: cystathionine β-synthase (CBS); cystathionine γ-lyase (CSE), and 3-mercapto-pyruvate sulfurtransferase (MST), which works together with cysteine aminotransferase (CAT) (24, 25). Cysteine-derived organic compounds, such as pyruvate, α-ketobutyrate and glutamate (26), supply other metabolic pathways (Figure 1A), such as the tricarboxylic acid (TCA) cycle and glucose-related pathways. Besides organic compounds, cysteine catabolism generates hydrogen sulfide (H_2_S) (27–32). Thus, the role of the enzymes has been directly associated with ATP production, as H_2_S can donate electrons to electron transport chain (ETC) (27, 28, 33, 34), and indirectly with the role of H_2_S as a paracrine and an autocrine signaling molecule in cancer, regulating cell proliferation, bioenergetics and angiogenesis (35, 36). The link between the enzymes involved in cysteine degradation and malignancy (27–29, 37–42) is thereby not easy to distinguish as being specifically related to the release of H_2_S or to the generation of organic compounds.

Cysteine catabolism cannot be addressed without mentioning that *de novo* cysteine synthesis occurs through the transsulfuration pathway (TSP), deriving from methionine and serine (Figure 1B), which makes the synthesis of cysteine dependent on the availability of methionine cycle intermediates (43). Serine and glycine can be glutamine-originated, making an interconnection of glutamine and cysteine metabolism (3). In methionine cycle, homocysteine is synthesized, being further condensed with serine to generate cystathionine, by CBS. Afterwards cystathionine is hydrolyzed by CSE, giving rise to cysteine, and other compounds (e.g., ammonia, α-ketobutyrate or propionate) [as reviewed (44)].

Pyruvate kinase (PK) is considered a main regulator of energy homeostasis by the generation of glucose-derived pyruvate (45), but recently, cysteine catabolism and serine synthesis pathway (SSP) were considered the main supplier of pyruvate in cancer cells, as a way of overcoming the lack of PK expression (46).

### One-Carbon Metabolism Concurrently Depends on and Controls Cysteine Bioavailability

The one-carbon metabolism is constituted by the methionine cycle and the folate cycle, which are dependent on serine and glycine bioavailability and from which certain intermediates are deviated to form cysteine (Figure 1B). Serine is synthesized from glucose and glutamine, and in turn serine gives rise to glycine [as reviewed (47)], which enters the folate cycle (48). Interestingly, cancer cells produce glycine from serine rather than import glycine (49), pointing out the upregulation of SSP as a cancer specialization. Moreover, phosphoglycerate dehydrogenase, a SSP key enzyme, was recently proposed as a poor prognosis marker in lung (50), gastric (51), and pancreatic (52) carcinomas.

The folate cycle depends on the dietary folate and controls the systemic levels of methionine and homocysteine (53), which directly regulates cysteine bioavailability. This cycle uses glycine and tetrahydrofolate (THF; converted from folic acid) and produces intermediates [5,10-methylene-tetrahydrofolate (5,10-MTHF) and 5-methylene-tetrahydrofolate (5-MTHF)] to supply purine synthesis and afterwards by the entrance of cobalamin (vitamin B_12_) and the interconnection with the methionine cycle, folic acid is again synthesized (Figure 1B).

The import of methionine is a vital step in one carbon metabolism, since methionine is an essential amino acid (AA), which is sequentially converted into S-adenosylmethionine (SAM) and S-adenosylhomocysteine (SAH), releasing methyl groups (CH_3_) that will be used in DNA, RNA, and histones methylation (Figure 1B). SAH can be deviated to originate pyrimidines or originate homocysteine, which will react with vitamin B_12_ and 5-MTHF in order to resynthesize methionine. Homocysteine can be deviated from one carbon metabolism and, together with serine, enter in TSP to originate cysteine and propionyl-CoA under the action of CBS and CSE (21, 54). Propionyl-CoA can be further converted into AA, fatty acids (FA) and TCA cycle intermediates (22, 23).

Methionine scarcity impairs cancer cells' proliferation (55), and methionine dependency is controlled by PI3K/AKT/mTOR pathway through the induction of the expression of cyst(e)ine/glutamate antiporter xc- (xCT; *SLC7A11* gene) (56), ensuring that the levels of cysteine won't limit the bioavailability of methionine, since cysteine uptake downregulates TSP.

As above mentioned, the methyl groups generated in the one-carbon metabolism, when released from methionine cycle, are crucial for DNA, RNA, and histones methylation for epigenetic modulation (57), whose functioning is regulated by PI3K/mTOR and HIF2α pathways, the same that control SSP and one-carbon metabolism (58, 59). Hence, the expression of LAT1 (*SLC7A5*), the main transporter of methionine, is associated with the activity of methyltransferases in lung cancer cells (60). Moreover, the relevance of one carbon metabolism is also highlighted by the association between the levels of folate in peripheral blood, DNA methylation and colorectal tumor staging (61). Accordingly, the existence of polymorphisms and the increased expression or activity of enzymes participating in one-carbon metabolism are considered markers for highly proliferative and aggressive cancer phenotypes and chemoresistance (57, 62, 63).

### Cysteine Contribution for Gluconeogenesis and Phosphate Pentose Pathway (PPP)

Gluconeogenesis or the synthesis of glucose from non-glucidic compounds, such as glycerol, lactate, pyruvate, acetyl-CoA, or glucogenic AA, only recently started to be explored in cancer. Gluconeogenesis (Figure 1C) is a reversion of glycolysis, with 3 alternative reactions counteracting the 3 irreversible steps of glycolysis (64–67). Cysteine is a glucogenic AA, as it originates pyruvate, however, as far as I know, cysteine was not yet explored as a source of glucose in cancer. Nevertheless, in other biological models cysteine has been pointed out as an important regulator of enzymes, such as peroxidases that can interact with PK and block the conversion of pyruvate into acetyl-CoA, avoiding pyruvate entrance in TCA cycle or in FA synthesis (68) and favoring its deviation into gluconeogenesis, ensuring the cell needs of glucose.

Gluconeogenic enzymes are regulated by signaling pathways pivotal in carcinogenesis KRAS-dependent, PIK3/mTOR and Wnt pathways and HIF1 [as reviewed in (69) and in (70)]. The pro-survival character of gluconeogenesis is supported by the upregulation or the *de novo* expression of its enzymes in different cancer types, such as breast, colon, stomach, uterine cervix, liver, and pancreas (67).

The inhibition of the final step of gluconeogenesis redirects glucose 6-phosphate to phosphate pentose pathway (PPP) (Figure 1C), making gluconeogenesis a supplier of PPP in glucose depleted environments. Again, cysteine as a source of pyruvate can be at the origin of glucose-6-phosphate canalized to PPP.

The PPP occurs in parallel to glycolysis through two irreversible oxidative reactions followed by two biochemical branches (an oxidative and a non-oxidative branch) of reversible reactions (71). The non-oxidative branch of PPP (Figure 1C) uses glucose-6-phosphate to generate pentose phosphates for AA and nucleotides synthesis. While the oxidative branch of PPP generates NADPH, essential for FA synthesis and redox balance (72–75). Indeed, a cellular dependence on PPP was described in cancer cells that are heavy cystine importers, requiring NADPH for cystine to cysteine intracellular conversion (76).

PPP is associated with increased cancer cell survival and proliferation (74, 77), implying the inhibition of phosphofructokinase (mainly PFK1) from glycolysis (78, 79), a direct competitor of glucose-6-phosphate dehydrogenase (G6PD), the limiting enzyme in PPP (75). PI3K/AKT pathway controls the expression and the activity of G6PD, whose dimerization is activated by phosphorylation (80). Wnt/c-MYC and p65-NFkB pathways induce the expression of G6PD, activating PPP as part of a more metastatic and chemoresistant cancer phenotype (81, 82).

Besides cysteine is a source of pyruvate, another important link of gluconeogenesis and PPP to cysteine metabolism and anti-oxidant character (83), is the fact that the expression of PCK1 (phosphoenolpyruvate carboxykinase 1) and G6PD is directly regulated by Nrf2, a master regulator of redox control (84, 85).

### Regulation of Cysteine Anabolism and Catabolism, in Cancer

The metabolic reliance on cysteine is a common feature to different cancer types. Therefore, the upregulation of catabolic pathways and the expression of cyst(e)ine transporters is often observed in cancer together with the upregulation of cysteine synthesis. The TSP is dependent on the action of CBS and CSE, which can also act in cysteine catabolism (Figure 1B). The expression of CBS and CSE seems to be cancer type-related often dependent on the organ and the genetic background.

In ovaries, it seems that CSE must be silenced upon malignant transformation, since it is expressed in normal epithelial ovarian cells but it is absent in malignant tumors (27, 86). On the contrary, the high CBS expression is a feature of ovarian cancer, being associated with advanced stage and chemoresistance (27, 86). In colon cancer, the increased expression and activity of CBS and CSE is associated with high rates of proliferation and migration of cancer cells, controlled, respectively, by PI3K/AKT and Wnt pathways (28, 86, 87). Controversially to the evidence that CBS is linked to carcinogenesis, a study presents CBS as a tumor suppressor gene, claiming that in gastric and colorectal cancer the expression of CBS is inhibited by DNA methylation in association with KRAS mutations (88). Notwithstanding a study reporting the importance of both CBS and CSE in gastric carcinogenesis (89), other study shows a compensatory mechanism involving the two enzymes. It was demonstrated that CSE expression overlaps the absence of CBS, being CSE correlated to increased proliferation and decreased apoptotic rate (41). In thyroid cancer, CBS is the major responsible for H_2_S production, which activates cancer cells proliferation and migration, through ROS/PI3K/AKT/mTOR and MAPK pathways (90). In breast cancer, tumors, and cell lines, CSE favors cell proliferation and migration under the command of STAT3, a member of JAK/STAT pathway (38); while in a murine model, CSE is stated as controlling the metastatic behavior of breast cancer cells through VEGF-dependent PI3K and MAPK pathways (91). In melanoma, CSE loss of expression accompanies the progression of the disease, being highly expressed in primary tumors and low expressed in metastatic lesions (30). The abovementioned data supports that the role of CBS and CSE enzymes, favoring or counteracting cancer, is highly adaptive and obviously dependent on the cysteine bioavailability itself, within certain cancer microenvironmental and metabolic contexts. Furthermore, if the role of CBS and CSE in cancer is related to cysteine anabolism or catabolism is not always clear.

Cysteine degradation catalyzed by CAT and MST (Figure 1B) is not deeply explored in cancer, since MST is more enzymatically efficient at a pH higher than the physiological, thus the role of CBS and CSE is considered more relevant in cancer biology (92). However, Zuhra et al. (93) demonstrated recently, in a colon cancer cell line, that MST can produce H_2_S from N-acetylcysteine instead of cysteine-derived 3-mercaptopyruvate. Nonetheless, MST is constitutively expressed in normal differentiated cells and some studies have detected its expression or activity in various cancer cell lines and primary tumors, including brain, colon, liver, kidney, lung and bladder cancer, and melanoma [reviewed in (35)]. In some of those studies the MST expression was higher than CSE expression (94, 95), and an association between MST expression and chemoresistance was found (96, 97). Few functional assays tried to correlate the expression and/or activity of MST with the cancer cells features, however, using inhibiting and silencing assays, some studies proved that MST activity is important for cancer cells proliferation (98, 99). Unfortunately, most studies addressing cysteine degradation are focused in H_2_S production and not in resulting organic compounds.

## Regulators and Mediators of Cysteine Transport, in Cancer

The transport of cysteine across the cell membrane is a critical step in cysteine metabolic course (Figure 1), and it is often transported in its oxidized dimer, cystine. Amongst cystine transporters, the cystine/glutamate antiporters are the most studied in cancer context, but mainly on their role in glutamate export (100, 101), showing a correlation between glutamate export and increased cancer cells aggressiveness (100, 102–106). However, for glutamate export to occur cystine import is mandatory, thus the increased intracellular levels of cysteine must be relevant for cancer poor prognosis. This evidence is reinforced by the activation of cysteine endogenous synthesis (56, 107) in cancer cells upon xCT downregulation (108–110).

xCT is an undeniable linker between cysteine and the whole metabolic network. Cancer cells overexpressing xCT present an overactivation of the glucose-dependent PPP, as a mean of replacing NADPH consumed in the imperative conversion of cystine into cysteine (76). Furthermore, xCT makes a bridge between cysteine uptake and glutamine metabolism, since glutamine is the main precursor of glutamate, whose export is essential for xCT-mediated import of cysteine (17). The role of xCT, as a facilitator of cyst(e)ine protective antioxidant role in cancer cells, is evidenced by the regulation of its expression by Nrf2 (111) and by signaling pathways activated by oxidative stress, including PI3K/AKT/mTOR (56, 112, 113) and MAPK pathways (110). Since augmented glutathione contributes for chemoresistance, the expression of xCT is also associated with resistance to drugs, platinum-salts (9) and epigenetic modulators (114), and with cell death evasion (115, 116). Considering a new cell death process, called ferroptosis, xCT is an important inhibitor, since the accumulation of lipid peroxides activates ferroptosis and cysteine-derived glutathione is the substrate used by glutathione peroxidase 4 (GPX4) in the dissipation of lipids peroxides (117).

The cysteine direct import (118) is mediated by cysteine transporters, and the expression of some of them have been addressed in cancer. Albeit, the promiscuity of these transporters in transferring different AA (e.g., cysteine, glutamine, and glutamate) impedes the direct association between their overexpression and cysteine uptake. Even though, their expression is relevant in cancer as it happens with AT-B^0, +^ (*SLC6A14*), which is the transporter with the broadest selectivity for AA, including cysteine (119–123).

In brief, EAAT3 (*SLC1A1*) overexpression was detected in brain and prostate cancer cells (124–126), being associated with increased chemoresistance in colorectal cancer models (127). As mentioned above, LAT1 can affect the bioavailability of cysteine since it is the main methionine transporter, being its expression related to chemoresistance (128). ASCT1 (*SLC1A4*) is overexpressed in prostate cancer (129), however, its expression and relevance in cancer was addressed considering glutamine or glutamate transport. Because glutamine/glutamate and cysteine metabolic pathways are deeply connected (130, 131) and cysteine is also considered a modulator of glutamine transport (132, 133), certainly these transporters are crucial in the cysteine metabolism reliance of cancer cells.

## Discussion

**Figure 2:**
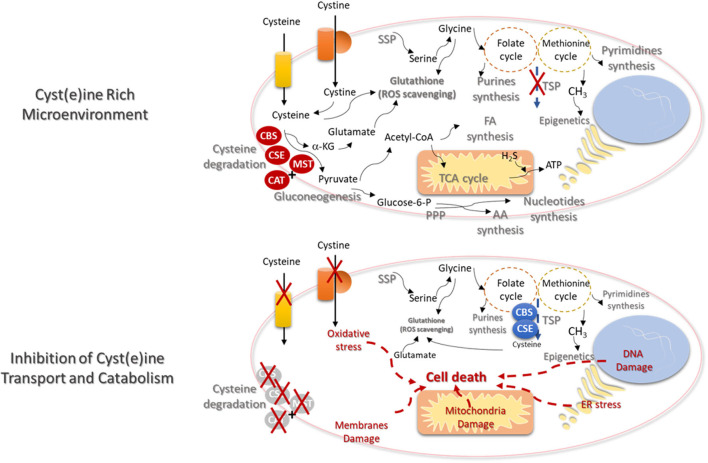
Cysteine transport and catabolism ensures cell functioning- new cues on metabolism based therapies. In a cyst(e)ine rich tumor microenvironment, cancer cells express high levels of cyst(e)ine transporters and the catabolism of cysteine is activated by the action of cystathionine β-synthase (CBS); cystathionine γ-lyase (CSE), or 3-mercapto-pyruvate sulfurtransferase (MST), which works together with cysteine aminotransferase (CAT). *De novo* synthesis of cysteine (transsulfuration pathway—TSP) will be diminished and the syntheses of serine (serine synthesis pathway—SSP) and glycine will supply the one-carbon metabolism (folate and methionine cycles), in order to support the synthesis of nucleotides and methyl groups, respectively, needed for cell proliferation and epigenetic regulation. Serine-derived glycine together with cysteine and glutamate constitute the glutathione molecule, crucial for the maintenance of the redox state needed for cellular metabolic functioning. Glutamate itself can be cysteine-derived, since α-ketoglutarate (α-KG) results from cysteine degradation and is directly converted into glutamate. Cysteine as a source of pyruvate contributes for biomass production, through the TCA cycle and the syntheses of FA and AA. Cysteine-derived pyruvate can be a substrate to produce glucose through gluconeogenesis, making a bridge between cysteine and glucose-dependent pathways, as phosphate pentose pathway (PPP). Electron donors generated in the metabolic pathways and cysteine-derived H_2_S contributes to oxidative phosphorylation and ATP production. The inhibition of cysteine uptake and catabolism will affect the metabolic pathways dependent on cysteine. TSP will be activated but without the uptake of cysteine, the ability to maintain the glutathione levels and reactive oxygen species (ROS) scavenging capacity will be decreased. The augment of the oxidative stress will induce DNA, membranes and mitochondria damages and endoplasmic reticulum (ER) stress. Ultimately cell injury and death will be triggered.

The increased intracellular bioavailability of cysteine is itself a stimulus for metabolic remodeling. Considering the role of cysteine as a carbon source in a scenario of high concentrations of cysteine, with no limitation in cyst(e)ine uptake, most part of cysteine will enter the degradation route, reducing the need for cysteine synthesis, dependent on the deviation of homocysteine from the one-carbon metabolism (Figure 2). This would imply the accumulation of serine that is very important for glycine synthesis and the activity of folate and methionine cycles, in order to supply the synthesis of nucleotides and methyl groups, respectively, needed for cell proliferation and epigenetic regulation. Serine-derived glycine together with cysteine and glutamate constitute the glutathione molecule, essential for the maintenance of the redox state allowing cellular metabolic functioning and chemoresistance. Glutamate can be a product of cysteine conversion into pyruvate, with α-ketoglutarate consume. Further, glutamate can be converted into glutamine, which is considered the main substitute of glucose (134).

Cysteine as a source of pyruvate can liberate the cell from the dependency of glucose, and contribute for biomass production, through the TCA cycle and the syntheses of FA and AA. As aforementioned, cysteine-derived pyruvate can be a substrate to produce glucose, making a bridge between cysteine and glucose-dependent pathways, as glycolysis and PPP. All the metabolic pathways that generate electron donors participating in the oxidative phosphorylation can be supplied by cysteine. In another hand, cysteine degradation releases H_2_S, which is itself an electron donor for ETC.

In brief, cysteine metabolic route is full of cues to find biomarkers for prognosis, recurrence and response to therapy, as well as suitable therapeutic targets to trigger cancer cell death due to cysteine starvation (Figure 2), as pointed out in different papers (135, 136). In certain type of cancer, it may be an unsuccessful strategy, since many cancer cells upon cysteine scarcity or the inhibition of cys(e)ine transport can upregulate TSP for endogenous cysteine production (137, 138). However, the need of methyl groups for epigenetic regulation, in some tumors, prevents the activation of cysteine synthesis and activates one-carbon metabolism (138). Therefore, the systemic decrease of cysteine levels is proposed as a suitable strategy in cancer clinical management, being supported by pre-clinical studies with promising results in breast and prostate carcinomas and leukemia. These studies showed that systemic treatment with cyst(e)inase decreases the levels of cysteine together with tumor burden (139). Cyst(e)inase degrades extracellular cysteine and cystine, leading to reduced intracellular cysteine and glutathione levels, affecting cancer cells redox capacity (44, 140), inducing the accumulation of ROS (26) and consequent ferroptosis (135, 136).

This review has also the objective of highlighting that efforts must be made to clarify the actual role of cysteine catabolism in cancer biosynthesis and bioenergetics, beyond H_2_S production. Cysteine catabolism may not be a core metabolic pathway but deviation of cysteine-derived compounds into other metabolic pathways is pivotal in cancer cells metabolic drift and survival.

## Author Contributions

The author confirms being the sole contributor of this work and has approved it for publication.

## Conflict of Interest

The author declares that the research was conducted in the absence of any commercial or financial relationships that could be construed as a potential conflict of interest.

## References

[1] Nunes SC Lopes-Coelho F Gouveia-Fernandes S Ramos C Pereira SA Serpa J. Cysteine boosters the evolutionary adaptation to CoCl(2) mimicked hypoxia conditions, favouring carboplatin resistance in ovarian cancer. BMC Evol Biol. (2018) 18:97. 10.1186/s12862-018-1214-1 29921232 PMC6011206

[2] Nunes SC Ramos C Lopes-Coelho F Sequeira CO Silva F Gouveia-Fernandes S. Cysteine allows ovarian cancer cells to adapt to hypoxia and to escape from carboplatin cytotoxicity. Sci Rep. (2018) 8:9513. 10.1038/s41598-018-27753-y 29934500 PMC6015047

[3] Lopes-Coelho F Gouveia-Fernandes S Gonçalves LG Nunes C Faustino I Silva F. HNF1β drives glutathione (GSH) synthesis underlying intrinsic carboplatin resistance of ovarian clear cell carcinoma (OCCC). Tumor Biol. (2016) 37:4813–29. 10.1007/s13277-015-4290-5 26520442

[4] Colla R Izzotti A De Ciucis C Fenoglio D Ravera S Speciale A. Glutathione-mediated antioxidant response and aerobic metabolism: two crucial factors involved in determining the multi-drug resistance of high-risk neuroblastoma. Oncotarget. (2016) 7:70715–37. 10.18632/oncotarget.12209 27683112 PMC5342585

[5] Zanotto-Filho A Masamsetti VP Loranc E Tonapi SS Gorthi A Bernard X. Alkylating agent-induced NRF2 blocks endoplasmic reticulum stress-mediated apoptosis via control of glutathione pools and protein thiol homeostasis. Mol Cancer Ther. (2016) 15:3000–14. 10.1158/1535-7163.MCT-16-0271 27638861 PMC5136348

[6] Lien EC Lyssiotis CA Juvekar A Hu H Asara JM Cantley LC. Glutathione biosynthesis is a metabolic vulnerability in PI(3)K/Akt-driven breast cancer. Nat Cell Biol. (2016) 18:572–8. 10.1038/ncb3341 27088857 PMC4848114

[7] Harris Isaac S Treloar Aislinn E Inoue S Sasaki M Gorrini C Lee Kim C. Glutathione and Thioredoxin Antioxidant Pathways Synergize To Drive Cancer Initiation And Progression. Cancer Cell. (2015) 27:211–22. 10.1016/j.ccell.2014.11.019 25620030

[8] Traverso N Ricciarelli R Nitti M Marengo B Furfaro AL Pronzato MA. Role of glutathione in cancer progression and chemoresistance. Oxid Med Cell Longev. (2013) 2013:972913–. 10.1155/2013/972913 23766865 PMC3673338

[9] Okuno S Sato H Kuriyama-Matsumura K Tamba M Wang H Sohda S. Role of cystine transport in intracellular glutathione level and cisplatin resistance in human ovarian cancer cell lines. Br J Cancer. (2003) 88:951–6. 10.1038/sj.bjc.6600786 12644836 PMC2377069

[10] Ballatori N Krance SM Notenboom S Shi S Tieu K Hammond CL. Glutathione dysregulation and the etiology and progression of human diseases. Biol Chem. (2009) 390:191–214. 10.1515/BC.2009.033 19166318 PMC2756154

[11] Wu G Fang Y-Z Yang S Lupton JR Turner ND. Glutathione metabolism and its implications for health. J Nutr. (2004) 134:489–92. 10.1093/jn/134.3.489 14988435

[12] Wang W Ballatori N. Endogenous glutathione conjugates: occurrence and biological functions. Pharmacol Rev. (1998) 50:335–56. 9755286

[13] Kalinina EV Chernov NN Novichkova MD. Role of glutathione, glutathione transferase, and glutaredoxin in regulation of redox-dependent processes. Biochem Biokhimiia. (2014) 79:1562–83. 10.1134/S0006297914130082 25749165

[14] Sosa V Moline T Somoza R Paciucci R Kondoh H Lleonart ME. Oxidative stress and cancer: an overview. Ageing Res Rev. (2013) 12:376–90. 10.1016/j.arr.2012.10.004 23123177

[15] Postovit L Widmann C Huang P Gibson SB. Harnessing oxidative stress as an innovative target for cancer therapy. Oxid Med Cell Longev. (2018) 2018:6135739. 10.1155/2018/6135739 29977457 PMC5994291

[16] Prasad S Srivastava SK. Oxidative stress and cancer: chemopreventive and therapeutic role of triphala. Antioxidants. (2020) 9:72. 10.3390/antiox9010072 31941067 PMC7022920

[17] Serpa J. Metabolic remodeling as a way of adapting to tumor microenvironment (TME), a job of several holders. Adv Exp Med Biol. (2020) 1219:1–34. 10.1007/978-3-030-34025-4_1 32130691

[18] Lin X Xiao Z Chen T Liang SH Guo H. Glucose metabolism on tumor plasticity, diagnosis, and treatment. Front Oncol. (2020) 10:317. 10.3389/fonc.2020.00317 32211335 PMC7069415

[19] Zou S Shimizu T Shimizu S Higashi Y Nakamura K Ono H. Possible role of hydrogen sulfide as an endogenous relaxation factor in the rat bladder and prostate. Neurourol Urodyn. (2018) 37:2519–26. 10.1002/nau.23788 30095194

[20] Huang CW Moore PK. H_2_S synthesizing enzymes: biochemistry and molecular aspects. In: Moore PK Whiteman M editors. Chemistry, Biochemistry and Pharmacology of Hydrogen Sulfide. Cham: Springer International Publishing (2015). p. 3–25. 10.1007/978-3-319-18144-8_1 26162827

[21] Pascale RM Peitta G Simile MM Feo F. Alterations of methionine metabolism as potential targets for the prevention and therapy of hepatocellular carcinoma. Medicina. (2019) 55:296. 10.3390/medicina55060296 31234428 PMC6631235

[22] Longo N Price LB Gappmaier E Cantor NL Ernst SL Bailey C. Anaplerotic therapy in propionic acidemia. Mol Genet Metab. (2017) 122:51–9. 10.1016/j.ymgme.2017.07.003 28712602 PMC5612888

[23] Adeva-Andany MM López-Maside L Donapetry-García C Fernández-Fernández C Sixto-Leal C. Enzymes involved in branched-chain amino acid metabolism in humans. Amino Acids. (2017) 49:1005–28. 10.1007/s00726-017-2412-7 28324172

[24] Wang R. Physiological implications of hydrogen sulfide: a whiff exploration that blossomed. Physiol Rev. (2012) 92:791–896. 10.1152/physrev.00017.2011 22535897

[25] Liu M Wu L Montaut S Yang G. Hydrogen sulfide signaling axis as a target for prostate cancer therapeutics. Prostate Cancer. (2016) 2016:9. 10.1155/2016/8108549 27019751 PMC4785274

[26] Nunes SC Serpa J. Glutathione in ovarian cancer: a double-edged sword. Int J Mol Sci. (2018) 19:1882. 10.3390/ijms19071882 29949936 PMC6073569

[27] Bhattacharyya S Saha S Giri K Lanza IR Nair KS Jennings NB. Cystathionine beta-synthase (CBS) contributes to advanced ovarian cancer progression and drug resistance. PLoS ONE. (2013) 8:e79167. 10.1371/journal.pone.0079167 24236104 PMC3827285

[28] Szabo C Coletta C Chao C Módis K Szczesny B Papapetropoulos A. Tumor-derived hydrogen sulfide, produced by cystathionine-β-synthase, stimulates bioenergetics, cell proliferation, and angiogenesis in colon cancer. Proc Natl Acad Sci USA. (2013) 110:12474–9. 10.1073/pnas.1306241110 23836652 PMC3725060

[29] Sen S Kawahara B Gupta D Tsai R Khachatryan M Roy-Chowdhuri S. Role of cystathionine β-synthase in human breast cancer. Free Radic Biol Med. (2015) 86:228–38. 10.1016/j.freeradbiomed.2015.05.024 26051168

[30] Panza E De Cicco P Armogida C Scognamiglio G Gigantino V Botti G. Role of the cystathionine γ lyase/hydrogen sulfide pathway in human melanoma progression. Pigment Cell Melanoma Res. (2015) 28:61–72. 10.1111/pcmr.12312 25205294

[31] Gai J-W Qin W Liu M Wang H-F Zhang M Li M. Expression profile of hydrogen sulfide and its synthases correlates with tumor stage and grade in urothelial cell carcinoma of bladder. Urol Oncol Semi Original Investig. (2016) 34:166.e15–e20. 10.1016/j.urolonc.2015.06.020 26847849

[32] Pan Y Zhou C Yuan D Zhang J Shao C. Radiation exposure promotes hepatocarcinoma cell invasion through epithelial mesenchymal transition mediated by H_2_S/CSE pathway. Radiat Res. (2015) 185:96–105. 10.1667/RR14177.1 26727544

[33] Módis K Panopoulos P Coletta C Papapetropoulos A Szabo C. Hydrogen sulfide-mediated stimulation of mitochondrial electron transport involves inhibition of the mitochondrial phosphodiesterase 2A, elevation of cAMP and activation of protein kinase A. Biochem Pharmacol. (2013) 86:1311–9. 10.1016/j.bcp.2013.08.064 24012591

[34] Fu M Zhang W Wu L Yang G Li H Wang R. Hydrogen sulfide (H_2_S) metabolism in mitochondria and its regulatory role in energy production. Proc Natl Acad Sci USA. (2012) 109:2943–8. 10.1073/pnas.1115634109 22323590 PMC3287003

[35] Augsburger F Szabo C. Potential role of the 3-mercaptopyruvate sulfurtransferase (3-MST)—hydrogen sulfide (H_2_S) pathway in cancer cells. Pharmacol Res. (2020) 154:104083. 10.1016/j.phrs.2018.11.034 30500457

[36] Giuffrè A Vicente JB. Hydrogen sulfide biochemistry and interplay with other gaseous mediators in mammalian physiology. Oxid Med Cell Longev. (2018) 2018:6290931. 10.1155/2018/6290931 30050658 PMC6040266

[37] Wang L Shi H Zhang X Zhang X Liu Y Kang W. I157172, a novel inhibitor of cystathionine gamma-lyase, inhibits growth and migration of breast cancer cells via SIRT1-mediated deacetylation of STAT3. Oncol Rep. (2019) 41:427–36. 10.3892/or.2018.6798 30365149

[38] You J Shi X Liang H Ye J Wang L Han H. Cystathionine- γ-lyase promotes process of breast cancer in association with STAT3 signaling pathway. Oncotarget. (2017) 8:65677–86. 10.18632/oncotarget.20057 29029463 PMC5630363

[39] Turbat-Herrera EA Kilpatrick MJ Chen J Meram AT Cotelingam J Ghali G. Cystathione β-synthase is increased in thyroid malignancies. Anticancer Res. (2018) 38:6085–90. 10.21873/anticanres.12958 30396922 PMC7771238

[40] Alix-Panabières C Cayrefourcq L Mazard T Maudelonde T Assenat E Assou S. Molecular portrait of metastasis-competent circulating tumor cells in colon cancer reveals the crucial role of genes regulating energy metabolism and DNA repair. Clin Chem. (2017) 63:700–13. 10.1373/clinchem.2016.263582 28007957

[41] Sekiguchi F Sekimoto T Ogura A Kawabata A. Endogenous hydrogen sulfide enhances cell proliferation of human gastric cancer AGS cells. Biol Pharm Bull. (2016) 39:887–90. 10.1248/bpb.b15-01015 27150157

[42] Poisson LM Munkarah A Madi H Datta I Hensley-Alford S Tebbe C. A metabolomic approach to identifying platinum resistance in ovarian cancer. J Ovarian Res. (2015) 8:13. 10.1186/s13048-015-0140-8 25880539 PMC4396147

[43] Pérez-Miguelsanz J Vallecillo N Garrido F Reytor E Pérez-Sala D Pajares MA. Betaine homocysteine S-methyltransferase emerges as a new player of the nuclear methionine cycle. Biochim Biophys Acta Mol Cell Res. (2017) 1864:1165–82. 10.1016/j.bbamcr.2017.03.004 28288879

[44] Combs JA DeNicola GM. The non-essential amino acid cysteine becomes essential for tumor proliferation and survival. Cancers. (2019) 11:678. 10.3390/cancers11050678 31100816 PMC6562400

[45] Kim YK Hammerling U. The mitochondrial PKCdelta/retinol signal complex exerts real-time control on energy homeostasis. Biochim Biophys Acta Mol Cell Biol Lipids. (2020) 2020:158614. 10.1016/j.bbalip.2020.158614 31927141 PMC7347429

[46] Yu L Teoh ST Ensink E Ogrodzinski MP Yang C Vazquez AI. Cysteine catabolism and the serine biosynthesis pathway support pyruvate production during pyruvate kinase knockdown in pancreatic cancer cells. Cancer Metab. (2019) 7:13. 10.1186/s40170-019-0205-z 31893043 PMC6937848

[47] DeBerardinis RJ. Serine metabolism: some tumors take the road less traveled. Cell Metab. (2011) 14:285–6. 10.1016/j.cmet.2011.08.004 21907134 PMC3172581

[48] Amelio I Cutruzzolá F Antonov A Agostini M Melino G. Serine and glycine metabolism in cancer. Trends Biochem Sci. (2014) 39:191–8. 10.1016/j.tibs.2014.02.004 24657017 PMC3989988

[49] Labuschagne Christiaan F van den Broek Niels JF Mackay Gillian M Vousden Karen H Maddocks Oliver DK. Serine, but not glycine, supports one-carbon metabolism and proliferation of cancer cells. Cell Rep. (2014) 7:1248–58. 10.1016/j.celrep.2014.04.045 24813884

[50] Zhang B Zheng A Hydbring P Ambroise G Ouchida AT Goiny M. PHGDH defines a metabolic subtype in lung adenocarcinomas with poor prognosis. Cell Rep. (2017) 19:2289–303. 10.1016/j.celrep.2017.05.067 28614715

[51] Xian Y Zhang S Wang X Qin J Wang W Wu H. Phosphoglycerate dehydrogenase is a novel predictor for poor prognosis in gastric cancer. Onco Targets Ther. (2016) 9:5553–60. 10.2147/OTT.S105787 27660473 PMC5019466

[52] Song Z Feng C Lu Y Lin Y Dong C. PHGDH is an independent prognosis marker and contributes cell proliferation, migration and invasion in human pancreatic cancer. Gene. (2018) 642:43–50. 10.1016/j.gene.2017.11.014 29128633

[53] Nazki FH Sameer AS Ganaie BA. Folate: metabolism, genes, polymorphisms and the associated diseases. Gene. (2014) 533:11–20. 10.1016/j.gene.2013.09.063 24091066

[54] Kulkarni A Dangat K Kale A Sable P Chavan-Gautam P Joshi S. Effects of altered maternal folic acid, vitamin B12 and docosahexaenoic acid on placental global DNA methylation patterns in Wistar rats. PLoS ONE. (2011) 6:e17706. 10.1371/journal.pone.0017706 21423696 PMC3053375

[55] Hens JR Sinha I Perodin F Cooper T Sinha R Plummer J Methionine-restricted diet inhibits growth of MCF10AT1-derived mammary tumors by increasing cell cycle inhibitors in athymic nude mice. BMC Cancer. (2016) 16:349 10.1186/s12885-016-2404-0 27255182 PMC4891836

[56] Lien EC Ghisolfi L Geck RC Asara JM Toker A. Oncogenic PI3K promotes methionine dependency in breast cancer cells through the cystine-glutamate antiporter xCT. Sci Signal. (2017) 10:eaao6604. 10.1126/scisignal.aao6604 29259101 PMC5808948

[57] Nilsson R Nicolaidou V Koufaris C. Mitochondrial MTHFD isozymes display distinct expression, regulation, and association with cancer. Gene. (2019) 716:144032. 10.1016/j.gene.2019.144032 31377316

[58] Green NH Galvan DL Badal SS Chang BH LeBleu VS Long J. MTHFD2 links RNA methylation to metabolic reprogramming in renal cell carcinoma. Oncogene. (2019) 38:6211–25. 10.1038/s41388-019-0869-4 31289360 PMC8040069

[59] Zeng JD Wu WKK Wang HY Li XX. Serine and one-carbon metabolism, a bridge that links mTOR signaling and DNA methylation in cancer. Pharmacol Res. (2019) 149:104352. 10.1016/j.phrs.2019.104352 31323332

[60] Dann SG Ryskin M Barsotti AM Golas J Shi C Miranda M. Reciprocal regulation of amino acid import and epigenetic state through Lat1 and EZH2. EMBO J. (2015) 34:1773–85. 10.15252/embj.201488166 25979827 PMC4516430

[61] Ferrari A Torrezan GT Carraro DM Aguiar Junior S. Association of folate and vitamins involved in the 1-carbon cycle with polymorphisms in the methylenetetrahydrofolate reductase gene (MTHFR) and global DNA methylation in patients with colorectal cancer. Nutrients. (2019) 11:1368. 10.3390/nu11061368 31216671 PMC6627304

[62] Gustafsson Sheppard N Jarl L Mahadessian D Strittmatter L Schmidt A Madhusudan N. The folate-coupled enzyme MTHFD2 is a nuclear protein and promotes cell proliferation. Sci Rep. (2015) 5:15029. 10.1038/srep15029 26461067 PMC4602236

[63] Zhang X Tang J Shen N Ren K. A single-nucleotide polymorphism (rs1805087) in the methionine synthase (METH) gene increases the risk of prostate cancer. Aging. (2018) 10:2741–54. 10.18632/aging.101584 30337500 PMC6224252

[64] Tsai WW Matsumura S Liu W Phillips NG Sonntag T Hao E. ATF3 mediates inhibitory effects of ethanol on hepatic gluconeogenesis. Proc Natl Acad Sci USA. (2015) 112:2699–704. 10.1073/pnas.1424641112 25730876 PMC4352786

[65] Potts A Uchida A Deja S Berglund ED Kucejova B Duarte JA. Cytosolic phosphoenolpyruvate carboxykinase as a cataplerotic pathway in the small intestine. Am J Physiol Gastrointest Liver Physiol. (2018) 315:G249–58. 10.1152/ajpgi.00039.2018 29631378 PMC6139646

[66] Zhang Y Guan Q Liu Y Zhang Y Chen Y Chen J. Regulation of hepatic gluconeogenesis by nuclear factor Y transcription factor in mice. J Biol Chem. (2018) 293:7894–904. 10.1074/jbc.RA117.000508 29530977 PMC5961052

[67] Grasmann G Smolle E Olschewski H Leithner K. Gluconeogenesis in cancer cells - repurposing of a starvation-induced metabolic pathway? Biochimica et Biophysica Acta Reviews Cancer. (2019) 1872:24–36. 10.1016/j.bbcan.2019.05.006 31152822 PMC6894939

[68] Irokawa H Tachibana T Watanabe T Matsuyama Y Motohashi H Ogasawara A. Redox-dependent regulation of gluconeogenesis by a novel mechanism mediated by a peroxidatic cysteine of peroxiredoxin. Sci Rep. (2016) 6:33536. 10.1038/srep33536 27634403 PMC5025857

[69] Lao-On U Attwood PV Jitrapakdee S. Roles of pyruvate carboxylase in human diseases: from diabetes to cancers and infection. J Mol Med. (2018) 96:237–47. 10.1007/s00109-018-1622-0 29362846

[70] Wang Z Dong C. Gluconeogenesis in cancer: function and regulation of PEPCK, FBPase, and G6Pase. Trends Cancer. (2019) 5:30–45. 10.1016/j.trecan.2018.11.003 30616754

[71] Ramos-Martinez JI. The regulation of the pentose phosphate pathway: remember Krebs. Arch Biochem Biophys. (2017) 614:50–2. 10.1016/j.abb.2016.12.012 28041936

[72] Pavlova NN Thompson CB. The emerging hallmarks of cancer metabolism. Cell Metab. (2016) 23:27–47. 10.1016/j.cmet.2015.12.006 26771115 PMC4715268

[73] Stincone A Prigione A Cramer T Wamelink MM Campbell K Cheung E. The return of metabolism: biochemistry and physiology of the pentose phosphate pathway. Biol Rev Camb Philos Soc. (2015) 90:927–63. 10.1111/brv.12140 25243985 PMC4470864

[74] Patra KC Hay N. The pentose phosphate pathway and cancer. Trends Biochem Sci. (2014) 39:347–54. 10.1016/j.tibs.2014.06.005 25037503 PMC4329227

[75] Jin L Zhou Y. Crucial role of the pentose phosphate pathway in malignant tumors. Oncol Lett. (2019) 17:4213–21. 10.3892/ol.2019.10112 30944616 PMC6444344

[76] Liu X Olszewski K Zhang Y Lim EW Shi J Zhang X. Cystine transporter regulation of pentose phosphate pathway dependency and disulfide stress exposes a targetable metabolic vulnerability in cancer. Nat Cell Biol. (2020) 22:476–86. 10.1038/s41556-020-0496-x 32231310 PMC7194135

[77] Weber GF. Metabolism in cancer metastasis. Int J Cancer. (2016) 138:2061–6. 10.1002/ijc.29839 26355498

[78] Yi W Clark PM Mason DE Keenan MC Hill C Goddard WA. Phosphofructokinase 1 glycosylation regulates cell growth and metabolism. Science. (2012) 337:975–80. 10.1126/science.1222278 22923583 PMC3534962

[79] Wang H Nicolay BN Chick JM Gao X Geng Y Ren H. The metabolic function of cyclin D3-CDK6 kinase in cancer cell survival. Nature. (2017) 546:426–30. 10.1038/nature22797 28607489 PMC5516959

[80] Hong X Song R Song H Zheng T Wang J Liang Y. PTEN antagonises Tcl1/hnRNPK-mediated G6PD pre-mRNA splicing which contributes to hepatocarcinogenesis. Gut. (2014) 63:1635–47. 10.1136/gutjnl-2013-305302 24352616

[81] Yin X Tang B Li JH Wang Y Zhang L Xie XY. ID1 promotes hepatocellular carcinoma proliferation and confers chemoresistance to oxaliplatin by activating pentose phosphate pathway. J Exp Clin Cancer Res. (2017) 36:166. 10.1186/s13046-017-0637-7 29169374 PMC5701377

[82] Gao Y Yang Y Yuan F Huang J Xu W Mao B. TNFalpha-YAP/p65-HK2 axis mediates breast cancer cell migration. Oncogenesis. (2017) 6:e383. 10.1038/oncsis.2017.83 28945218 PMC5623908

[83] Riganti C Gazzano E Polimeni M Aldieri E Ghigo D. The pentose phosphate pathway: an antioxidant defense and a crossroad in tumor cell fate. Free Radic Biol Med. (2012) 53:421–36. 10.1016/j.freeradbiomed.2012.05.006 22580150

[84] Kowalik MA Guzzo G Morandi A Perra A Menegon S Masgras I. Metabolic reprogramming identifies the most aggressive lesions at early phases of hepatic carcinogenesis. Oncotarget. (2016) 7:32375–93. 10.18632/oncotarget.8632 27070090 PMC5078020

[85] Tebay LE Robertson H Durant ST Vitale SR Penning TM Dinkova-Kostova AT. Mechanisms of activation of the transcription factor Nrf2 by redox stressors, nutrient cues, and energy status and the pathways through which it attenuates degenerative disease. Free Radic Biol Med. (2015) 88:108–46. 10.1016/j.freeradbiomed.2015.06.021 26122708 PMC4659505

[86] Hellmich MR Szabo C. Hydrogen sulfide and cancer. Handb Exp Pharmacol. (2015) 230:233–41. 10.1007/978-3-319-18144-8_12 26162838 PMC4665975

[87] Szabo C Hellmich MR. Endogenously produced hydrogen sulfide supports tumor cell growth and proliferation. Cell Cycle. (2013) 12:2915–6. 10.4161/cc.26064 23974103 PMC3875657

[88] Zhao H Li Q Wang J Su X Ng KM Qiu T. Frequent epigenetic silencing of the folate-metabolising gene cystathionine-beta-synthase in gastrointestinal cancer. PLoS ONE. (2012) 7:e49683. 10.1371/journal.pone.0049683 23152928 PMC3496708

[89] Zhang L Qi Q Yang J Sun D Li C Xue Y. An anticancer role of hydrogen sulfide in human gastric cancer cells. Oxid Med Cell Longev. (2015) 2015:636410. 10.1155/2015/636410 26078811 PMC4442311

[90] Wu D Li J Zhang Q Tian W Zhong P Liu Z. Exogenous hydrogen sulfide regulates the growth of human thyroid carcinoma cells. Oxid Med Cell Longev. (2019) 2019:6927298. 10.1155/2019/6927298 31223424 PMC6541980

[91] Wang L Shi H Liu Y Zhang W Duan X Li M. Cystathionine-γ-lyase promotes the metastasis of breast cancer via the VEGF signaling pathway. Int J Oncol. (2019) 55:473–87. 10.3892/ijo.2019.4823 31173185 PMC6615928

[92] Masi Ad Ascenzi P. H_2_S: a “Double face” molecule in health and disease. BioFactors. (2013) 39:186–96. 10.1002/biof.1061 23233276

[93] Zuhra K Tomé CS Masi L Giardina G Paulini G Malagrinò F. N-acetylcysteine serves as substrate of 3-mercaptopyruvate sulfurtransferase and stimulates sulfide metabolism in colon cancer cells. Cells. (2019) 8:828. 10.3390/cells8080828 31382676 PMC6721681

[94] Jurkowska H Placha W Nagahara N Wróbel M. The expression and activity of cystathionine-γ-lyase and 3-mercaptopyruvate sulfurtransferase in human neoplastic cell lines. Amino Acids. (2011) 41:151–8. 10.1007/s00726-010-0606-3 20446008

[95] Bronowicka-Adamska P Bentke A Wrobel M. Hydrogen sulfide generation from l-cysteine in the human glioblastoma-astrocytoma U-87 MG and neuroblastoma SHSY5Y cell lines. Acta Biochimica Polonica. (2017) 64:171–6. 10.18388/abp.2016_1394 28291844

[96] Ostrakhovitch EA Akakura S Sanokawa-Akakura R Goodwin S Tabibzadeh S. Dedifferentiation of cancer cells following recovery from a potentially lethal damage is mediated by H_2_S–Nampt. Exp Cell Res. (2015) 330:135–50. 10.1016/j.yexcr.2014.09.027 25278485

[97] Untereiner AA Pavlidou A Druzhyna N Papapetropoulos A Hellmich MR Szabo C. Drug resistance induces the upregulation of H_2_S-producing enzymes in HCT116 colon cancer cells. Biochem Pharmacol. (2018) 149:174–85. 10.1016/j.bcp.2017.10.007 29061341 PMC5866167

[98] Oláh G Módis K Törö G Hellmich MR Szczesny B Szabo C. Role of endogenous and exogenous nitric oxide, carbon monoxide and hydrogen sulfide in HCT116 colon cancer cell proliferation. Biochem Pharmacol. (2018) 149:186–204. 10.1016/j.bcp.2017.10.011 29074106 PMC5866187

[99] Szczesny B Marcatti M Zatarain JR Druzhyna N Wiktorowicz JE Nagy P. Inhibition of hydrogen sulfide biosynthesis sensitizes lung adenocarcinoma to chemotherapeutic drugs by inhibiting mitochondrial DNA repair and suppressing cellular bioenergetics. Sci Rep. (2016) 6:36125. 10.1038/srep36125 27808278 PMC5093586

[100] Lo M Wang Y-Z Gout PW. The x cystine/glutamate antiporter: a potential target for therapy of cancer and other diseases. J Cell Physiol. (2008) 215:593–602. 10.1002/jcp.21366 18181196

[101] Bianchi MG Bardelli D Chiu M Bussolati O. Changes in the expression of the glutamate transporter EAAT3/EAAC1 in health and disease. Cell Mol Life Sci. (2014) 71:2001–15. 10.1007/s00018-013-1484-0 24162932 PMC11113519

[102] Fazzari J Lin H Murphy C Ungard R Singh G. Inhibitors of glutamate release from breast cancer cells; new targets for cancer-induced bone-pain. Sci Rep. (2015) 5:8380. 10.1038/srep08380 25670024 PMC4323637

[103] Shiozaki A Iitaka D Ichikawa D Nakashima S Fujiwara H Okamoto K. xCT, component of cysteine/glutamate transporter, as an independent prognostic factor in human esophageal squamous cell carcinoma. J Gastroenterol. (2014) 49:853–63. 10.1007/s00535-013-0847-5 23771433

[104] Stepulak A Rola R Polberg K Ikonomidou C. Glutamate and its receptors in cancer. J Neural Transm. (2014) 121:933–44. 10.1007/s00702-014-1182-6 24610491 PMC4133641

[105] Koochekpour S Majumdar S Azabdaftari G Attwood K Scioneaux R Subramani D. Serum glutamate levels correlate with Gleason score and glutamate blockade decreases proliferation, migration, and invasion and induces apoptosis in prostate cancer cells. Clin Cancer Res. (2012) 18:5888–901. 10.1158/1078-0432.CCR-12-1308 23072969 PMC3492499

[106] Dornier E Rabas N Mitchell L Novo D Dhayade S Marco S. Glutaminolysis drives membrane trafficking to promote invasiveness of breast cancer cells. Nat Commun. (2017) 8:2255. 10.1038/s41467-017-02101-2 29269878 PMC5740148

[107] Kang ES Lee J Homma T Kurahashi T Kobayashi S Nabeshima A. xCT deficiency aggravates acetaminophen-induced hepatotoxicity under inhibition of the transsulfuration pathway. Free Rad Res. (2017) 51:80–90. 10.1080/10715762.2017.1282157 28081640

[108] Ji X Qian J Rahman SMJ Siska PJ Zou Y Harris BK. xCT (SLC7A11)-mediated metabolic reprogramming promotes non-small cell lung cancer progression. Oncogene. (2018) 37:5007–19. 10.1038/s41388-018-0307-z 29789716 PMC6127081

[109] Koppula P Zhang Y Shi J Li W Gan B. The glutamate/cystine antiporter SLC7A11/xCT enhances cancer cell dependency on glucose by exporting glutamate. J Biol Chem. (2017) 292:14240–9. 10.1074/jbc.M117.798405 28630042 PMC5572906

[110] Lim JKM Delaidelli A Minaker SW Zhang H-F Colovic M Yang H. Cystine/glutamate antiporter xCT (SLC7A11) facilitates oncogenic RAS transformation by preserving intracellular redox balance. Proc Natl Acad Sci USA. (2019) 116:9433–42. 10.1073/pnas.1821323116 31000598 PMC6511045

[111] Habib E Linher-Melville K Lin H-X Singh G. Expression of xCT and activity of system xc(-) are regulated by NRF2 in human breast cancer cells in response to oxidative stress. Redox Biol. (2015) 5:33–42. 10.1016/j.redox.2015.03.003 25827424 PMC4392061

[112] Mossmann D Park S Hall MN. mTOR signalling and cellular metabolism are mutual determinants in cancer. Nat Rev Cancer. (2018) 18:744–57. 10.1038/s41568-018-0074-8 30425336

[113] Gu Y Albuquerque CP Braas D Zhang W Villa GR Bi J. mTORC2 regulates amino acid metabolism in cancer by phosphorylation of the cystine-glutamate antiporter xCT. Mol Cell. (2017) 67:128–38.e7. 10.1016/j.molcel.2017.05.030 28648777 PMC5521991

[114] Miyamoto K Watanabe M Boku S Sukeno M Morita M Kondo H. xCT inhibition increases sensitivity to vorinostat in a ROS-dependent manner. Cancers. (2020) 12:827. 10.3390/cancers12040827 32235498 PMC7226257

[115] Yu H Yang C Jian L Guo S Chen R Li K. Sulfasalazine-induced ferroptosis in breast cancer cells is reduced by the inhibitory effect of estrogen receptor on the transferrin receptor. Oncol Rep. (2019) 42:826–38. 10.3892/or.2019.7189 31173262

[116] Li Y Yan H Xu X Liu H Wu C Zhao L. Erastin/sorafenib induces cisplatin-resistant non-small cell lung cancer cell ferroptosis through inhibition of the Nrf2/xCT pathway. Oncol Lett. (2020) 19:323–33. 10.3892/ol.2019.11066 31897145 PMC6923844

[117] Fujii J Homma T Kobayashi S. Ferroptosis caused by cysteine insufficiency and oxidative insult. Free Radic Res. (2019) 1–12. 10.1080/10715762.2019.1666983 31505959

[118] Zhang W Trachootham D Liu J Chen G Pelicano H Garcia-Prieto C. Stromal control of cystine metabolism promotes cancer cell survival in chronic lymphocytic leukaemia. Nat Cell Biol. (2012) 14:276–86. 10.1038/ncb2432 22344033 PMC3290742

[119] Coothankandaswamy V Cao S Xu Y Prasad PD Singh PK Reynolds CP. Amino acid transporter SLC6A14 is a novel and effective drug target for pancreatic cancer. Br J Pharmacol. (2016) 173:3292–306. 10.1111/bph.13616 27747870 PMC5738662

[120] Lieu EL Nguyen T Rhyne S Kim J. Amino acids in cancer. Exp Mol Med. (2020) 52:15–30. 10.1038/s12276-020-0375-3 31980738 PMC7000687

[121] Gupta N Miyauchi S Martindale RG Herdman AV Podolsky R Miyake K. Upregulation of the amino acid transporter ATB0,+ (SLC6A14) in colorectal cancer and metastasis in humans. Biochim Biophys Acta. (2005) 1741:215–23. 10.1016/j.bbadis.2005.04.002 15905073

[122] Gupta N Prasad PD Ghamande S Moore-Martin P Herdman AV Martindale RG. Up-regulation of the amino acid transporter ATB(0,+) (SLC6A14) in carcinoma of the cervix. Gynecol Oncol. (2006) 100:8–13. 10.1016/j.ygyno.2005.08.016 16168467

[123] Karunakaran S Umapathy NS Thangaraju M Hatanaka T Itagaki S Munn DH. Interaction of tryptophan derivatives with SLC6A14 (ATB0,+) reveals the potential of the transporter as a drug target for cancer chemotherapy. Biochem J. (2008) 414:343–55. 10.1042/BJ20080622 18522536

[124] Pissimissis N Papageorgiou E Lembessis P Armakolas A Koutsilieris M. The glutamatergic system expression in human PC-3 and LNCaP prostate cancer cells. Anticancer Res. (2009) 29:371–7. 19331175

[125] Bianchi MG Franchi-Gazzola R Reia L Allegri M Uggeri J Chiu M. Valproic acid induces the glutamate transporter excitatory amino acid transporter-3 in human oligodendroglioma cells. Neuroscience. (2012) 227:260–70. 10.1016/j.neuroscience.2012.09.055 23041758

[126] Aronica E Gorter JA Ijlst-Keizers H Rozemuller AJ Yankaya B Leenstra S. Expression and functional role of mGluR3 and mGluR5 in human astrocytes and glioma cells: opposite regulation of glutamate transporter proteins. Eur J Neurosci. (2003) 17:2106–18. 10.1046/j.1460-9568.2003.02657.x 12786977

[127] Pedraz-Cuesta E Christensen S Jensen AA Jensen NF Bunch L Romer MU. The glutamate transport inhibitor DL-Threo-β-Benzyloxyaspartic acid (DL-TBOA) differentially affects SN38- and oxaliplatin-induced death of drug-resistant colorectal cancer cells. BMC Cancer. (2015) 15:411. 10.1186/s12885-015-1405-8 25981639 PMC4445981

[128] Altan B Kaira K Watanabe A Kubo N Bao P Dolgormaa G. Relationship between LAT1 expression and resistance to chemotherapy in pancreatic ductal adenocarcinoma. Cancer Chemother Pharmacol. (2018) 81:141–53. 10.1007/s00280-017-3477-4 29149426

[129] White MA Lin C Rajapakshe K Dong J Shi Y Tsouko E. Glutamine transporters are targets of multiple oncogenic signaling pathways in prostate cancer. Mol Cancer Res. (2017) 15:1017–28. 10.1158/1541-7786.MCR-16-0480 28507054 PMC5685160

[130] Muir A Danai LV Gui DY Waingarten CY Lewis CA Vander Heiden MG. Environmental cystine drives glutamine anaplerosis and sensitizes cancer cells to glutaminase inhibition. Elife. (2017) 6:e27713. 10.7554/eLife.27713 28826492 PMC5589418

[131] Lian G Gnanaprakasam JR Wang T Wu R Chen X Liu L. Glutathione de novo synthesis but not recycling process coordinates with glutamine catabolism to control redox homeostasis and directs murine T cell differentiation. Elife. (2018) 7:e36158. 10.7554/eLife.36158 30198844 PMC6152796

[132] Scalise M Pochini L Console L Losso MA Indiveri C. The Human SLC1A5 (ASCT2) amino acid transporter: from function to structure and role in cell biology. Front Cell Dev Biol. (2018) 6:96. 10.3389/fcell.2018.00096 30234109 PMC6131531

[133] Scalise M Pochini L Pingitore P Hedfalk K Indiveri C. Cysteine is not a substrate but a specific modulator of human ASCT2 (SLC1A5) transporter. FEBS Lett. (2015) 589:3617–23. 10.1016/j.febslet.2015.10.011 26492990

[134] Gaglio D Metallo CM Gameiro PA Hiller K Danna LS Balestrieri C. Oncogenic K-Ras decouples glucose and glutamine metabolism to support cancer cell growth. Mol Syst Biol. (2011) 7:523. 10.1038/msb.2011.56 21847114 PMC3202795

[135] Conrad M Pratt DA. The chemical basis of ferroptosis. Nat Chem Biol. (2019) 15:1137–47. 10.1038/s41589-019-0408-1 31740834

[136] Zhou B Liu J Kang R Klionsky DJ Kroemer G Tang D Ferroptosis is a type of autophagy-dependent cell death. Semin Cancer Biol. (2019). 10.1016/j.semcancer.2019.03.002. [Epub ahead of print]. 30880243

[137] Liu N Lin X Huang C. Activation of the reverse transsulfuration pathway through NRF2/CBS confers erastin-induced ferroptosis resistance. Br J Cancer. (2020) 122:279–92. 10.1038/s41416-019-0660-x 31819185 PMC7052275

[138] Zhu J Berisa M Schworer S Qin W Cross JR Thompson CB. Transsulfuration activity can support cell growth upon extracellular cysteine limitation. Cell Metab. (2019) 30:865–76.e5. 10.1016/j.cmet.2019.09.009 31607565 PMC6961654

[139] Cramer SL Saha A Liu J Tadi S Tiziani S Yan W. Systemic depletion of L-cyst(e)ine with cyst(e)inase increases reactive oxygen species and suppresses tumor growth. Nat Med. (2017) 23:120–7. 10.1038/nm.4232 27869804 PMC5218918

[140] Kshattry S Saha A Gries P Tiziani S Stone E Georgiou G. Enzyme-mediated depletion of l-cyst(e)ine synergizes with thioredoxin reductase inhibition for suppression of pancreatic tumor growth. NPJ Prec Oncol. (2019) 3:16. 10.1038/s41698-019-0088-z 31231686 PMC6546752

